# Nonlinear Mixed-Effects Modelling of *In Vitro* Drug Susceptibility and Molecular Correlates of Multidrug Resistant *Plasmodium falciparum*

**DOI:** 10.1371/journal.pone.0069505

**Published:** 2013-07-24

**Authors:** Julie A. Simpson, Kris M. Jamsen, Tim J. C. Anderson, Sophie Zaloumis, Shalini Nair, Charles Woodrow, Nicholas J. White, Francois Nosten, Ric N. Price

**Affiliations:** 1 Centre for Molecular, Environmental, Genetic & Analytic Epidemiology, Melbourne School of Population and Global Health, University of Melbourne, Melbourne, Australia; 2 Texas Biomedical Research Institute, San Antonio, Texas, United States of America; 3 Centre for Tropical Medicine, Nuffield Department of Clinical Medicine, University of Oxford, Oxford, United Kingdom; 4 Mahidol-Oxford Tropical Medicine Research Unit, Bangkok, Thailand; 5 WorldWide Antimalarial Resistance Network (WWARN), Centre for Tropical Medicine, Nuffield Department of Clinical Medicine, University of Oxford, Oxford, United Kingdom; 6 Shoklo Malaria Research Unit, Mae Sot, Tak, Thailand; 7 Global Health Division, Menzies School of Health Research, Charles Darwin University, Darwin, Australia; Johns Hopkins Bloomberg School of Public Health, United States of America

## Abstract

The analysis of *in vitro* anti-malarial drug susceptibility testing is vulnerable to the effects of different statistical approaches and selection biases. These confounding factors were assessed with respect to *pfmdr1* gene mutation and amplification in 490 clinical isolates. Two statistical approaches for estimating the drug concentration associated with 50% effect (*EC_50_*) were compared: the commonly used standard two-stage (STS) method, and nonlinear mixed-effects modelling. The *in vitro* concentration-effect relationships for, chloroquine, mefloquine, lumefantrine and artesunate, were derived from clinical isolates obtained from patients on the western border of Thailand. All isolates were genotyped for polymorphisms in the *pfmdr1* gene. The *EC_50_* estimates were similar for the two statistical approaches but 15–28% of isolates in the STS method had a high coefficient of variation (>15%) for individual estimates of *EC_50_* and these isolates had *EC_50_* values that were 32 to 66% higher than isolates derived with more precision. In total 41% (202/490) of isolates had amplification of *pfmdr1* and single nucleotide polymorphisms were found in 50 (10%). *Pfmdr1* amplification was associated with an increase in *EC_50_* for mefloquine (139% relative increase in *EC_50_* for 2 copies, 188% for 3+ copies), lumefantrine (82% and 75% for 2 and 3+ copies respectively) and artesunate (63% and 127% for 2 and 3+ copies respectively). In contrast *pfmdr1* mutation at codons 86 or 1042 were associated with an increase in chloroquine *EC_50_* (44–48%). Sample size calculations showed that to demonstrate an *EC_50_* shift of 50% or more with 80% power if the prevalence was 10% would require 430 isolates and 245 isolates if the prevalence was 20%. In conclusion, although nonlinear mixed-effects modelling did not demonstrate any major advantage for determining estimates of anti-malarial drug susceptibility, the method includes all isolates, thereby, potentially improving confirmation of candidate molecular markers of anti-malarial drug susceptibility.

## Introduction

*In vitro* drug susceptibility assays provide a means of assessing anti-malarial drug activity. These assays are important tools for monitoring anti-malarial drug resistance, determining the relationship between parasite genetic polymorphisms and drug susceptibility, and quantifying the potency of novel compounds. A variety of methodological approaches have been developed and applied to assessment of laboratory adapted strains of Plasmodium as well as field isolates of *P. falciparum* and *P. vivax* [1]–[3]. Established assays vary, but in general define a parasites’ drug susceptibility by measurements of growth or development *ex vivo* in the constant presence of varying drug concentrations, usually generated by doubling dilutions of the drug under investigation. The standard statistical analysis of such assays focuses on fitting separate nonlinear regression equations to each isolate’s concentration-effect data, using a nonlinear Sigmoid Emax equation, deriving four measures: the minimum and maximum effect, the slope (sigmoidicity) of the effect-concentration curve, and the drug concentration that achieves 50% of the maximum effect (termed the *EC_50_*). The isolate-specific estimates of these parameters are then combined to calculate summary statistics that describe the population distribution. However this approach, referred to as the standard two-stage (STS) method [4], is vulnerable to a variety of selection biases [5]. Importantly the estimates of highly resistant isolates, often the most interesting parasites, are intrinsically less precise and thus often rejected [6]. In the case of laboratory isolates drug susceptibility assays can be repeated, however, in field studies repetition of the assay with revised drug concentrations is often not feasible from a fresh sample unless isolates are adapted to continuous culture, a process which may increase selection bias further. Simply excluding these extreme isolates as unreliable skews the population estimates towards a lower population mean *EC_50_*.

Nonlinear mixed-effects modelling is an alternative statistical approach for analysing repeated measurement data [7]. When applied to the analysis of *in vitro* drug susceptibility data, information from all of the isolates is analysed simultaneously. Estimates of the population mean values for each of the model parameters are derived, as well as the between-isolate variance of each parameter and the overall within-isolate variance. Although routinely used in sparsely sampled pharmacokinetic studies [8], [9], its application to the analysis of data from *in vitro* studies is limited.

On the western border of Thailand multidrug resistance in *P.falciparum* has been shown to correlate with polymorphisms of *pfmdr1*; gene amplification being associated with reduced sensitivity to mefloquine, lumefantrine and artemisinin derivatives [10], [11], whereas single nucleotide polymorphisms are associated with chloroquine resistance [12]. In this study, we have pooled a large collection of carefully characterised field isolates from Thailand, to review the *in vitro* – molecular correlates and intra-assay variation using the STS method and nonlinear mixed-effects modelling. The comparison is restricted to four anti-malarial drugs, chloroquine, mefloquine, lumefantrine and artesunate, to evaluate if the more complex and comprehensive method of nonlinear mixed-effects modelling, provides important additional information on the *in vitro* - molecular correlation of antimalarial drug resistance and specifically whether it provides increased statistical power for detecting shifts in *in vitro* susceptibility of subgroup parasite populations.

## Materials and Methods

### Clinical Isolates

Fresh parasite isolates were obtained from patients with acute *P. falciparum* malaria attending clinics of the Shoklo Malaria Research Unit (SMRU), between 1993 and 2005. The SMRU clinics are all located along 100 km of the Thai-Myanmar border. Isolates were collected from primary infections with a parasite density of at least 5 parasites/1,000 red blood cells. Venous blood (5 ml) was collected into a sterile Vacutainer® tube containing 0.05 ml Potassium-EDTA. Samples were kept at room temperature before being (within the next 4 to 6 hrs) transported to the main laboratory, where they were set up in continuous culture immediately. The fresh parasite isolate samples were obtained as part of prospective clinical evaluations of anti-malarial drug therapy. Written informed consent translated in the patient own language was obtained from each participant, whose signature was witnessed. The studies were approved by the Ethics Committee of the Faculty of Tropical Medicine, Mahidol University.

### In vitro Drug Assay

*In vitro* drug susceptibility was determined by the hypoxanthine uptake inhibition assay, the details of which have been described previously [13]. Briefly, fresh isolates were adjusted to an optimum density of 0.5–1.0% IRBC and a haematocrit of 1.5% using fresh washed group O erythrocytes and complete RPMI-1640 medium with 10% heat-inactivated AB sera. The suspension of infected erythrocytes was dispensed into the wells of a standard microtitre plate containing duplicate serial dilutions of the antimalarial drugs. Serial dilutions for the majority of isolates were measured as the following: 1646.6 to 1.62 nM for mefloquine, 87.0 to 0.044 nM for artesunate, 10255.9 to 10.02 nM for chloroquine, 235.8 to 2.40 nM for lumefantrine; and were made in complete RPMI medium. All drug concentrations, including drug-free controls, were generated in duplicate in 96-well tissue culture plates. The drug-plates were made in bulk and stored at −80°C until use. Following incubation for 24 h, the microtitre plates were pulsed with [3H] hypoxanthine isotopic solution to each well. After a further 18 h incubation, the plates were then harvested. The reproducibility of the *EC_50_* measurements was assessed regularly using cloned K1 isolates of *P.falciparum*.

### Molecular Analysis of pfmdr1

Details of the methods used to determine *pfmdr1* copy number have been described previously [14]. In summary, *pfmdr1* copy number was assessed by quantitative PCR (ABI sequence detector 7700 or 7900HT; Applied Biosystems™) and all reactions were performed in triplicate or quadruplicate. DNA for the molecular analysis of *pfmdr1* copy number was available from whole blood or from 50 µL of capillary blood transferred to filter paper. Genetic variants of *pfmdr1* occur either through single nucleotide polymorphisms at key loci or amplification of the whole gene resulting in increased copy number. In Thai isolates the latter process occurs almost exclusively in parasites of the wild type *pfmdr1* [11], [15]. For this reason parasites were classified into the five genotypes: 1) Single copy with the wild type allele including 86N and 1042N; 2) Single copy number with the 86Y mutation alone, 3) Single copy number with 1042D mutation; 4) Double copy number (all 86N and 1042N), 5) Triple or more copy number (also all 86N and 1042N).

### Statistical Analysis

The initial data set included all assays in which both *in vitro* and molecular data were available. The analysis was restricted to four anti-malarial drugs: chloroquine, mefloquine, lumefantrine and artesunate.

The nonlinear equation fitted to the data was the following sigmoid inhibitory effect model:
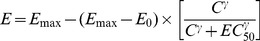
(1)


*E* represents the percentage of uptake of hypoxanthine and was normalized to the control wells using the following equation:
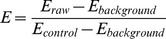



*E_raw_* is the absolute value for the uptake of hypoxanthine in the drug well, compared to that in the control well (*E_control_*) and that of the background radiation (*E_background_* ) from two wells containing only infected red blood cells. Maximum inhibition occurs when there is no uptake of hypoxanthine (no growth), that is, *E* equals 0. In equation 1, *E*_0_ represents minimum percent growth, *E*_max_ is the maximum percent growth, *EC*_50_ is the concentration of the drug required to inhibit 50 percent of the control parasites’ hypoxanthine uptake, *C* represents the drug concentration and *γ* is the slope of the curve.

Initially, the data were analysed using the STS method. For stage 1, the sigmoid inhibitory effect model (equation 1) was fitted to each individual isolate’s effect-concentration data by the application of nonlinear regression using the software package WinNonlin (Pharsight™ Corporation).

Isolates with predicted curves where the maximum uptake of hypoxanthine was greater than 125% or less than 75% and/or the minimum uptake of hypoxanthine was greater than 25% or less than −25% were excluded from all further analyses. Although some laboratories exclude isolates in which the coefficient of variation for the *EC_50_* (i.e. standard error divided by *EC_50_* estimate from nonlinear regression of effect-concentration curve) is greater than 15%, in the current study these isolates were included in the overall analysis.

The individual isolate estimates of *EC_50_* and slope (γ), and the coefficients of variation (standard error divided by estimate) for each estimate were saved in a data file (see File S1). For stage 2 of the analysis, the distribution of *EC_50_* and slope for the study population were summarised by calculating the geometric mean and 95% reference range of the individual estimates of *EC_50_* and slope.

Second, the data were analysed using nonlinear mixed-effects modelling. Effect-concentration data from all isolates were analysed simultaneously using equation 1, where for isolate *i* and drug concentration *j,* the sigmoid inhibitory effect *E*_ij_ was given as:
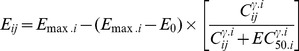
(2)where



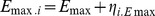


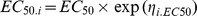


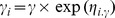

*E*_max,_ *EC*_50_ and γ represent the population means and η_i, *Emax*,_ η_i, *EC*50_ and η_i, *γ*_ are normally distributed random-effects with mean zero and variances Ω*_Emax_*_,_ Ω*_EC_*_50_ and Ω*_γ_*, respectively. Thus the Ωs represent the between-isolate variability for these parameters, reflecting a variety of confounders, including methodological, host and parasite factors that may retain inherent influence within the assay. Between-isolate variability in *EC*_50_ and γ was assumed to be lognormally distributed; a reasonable assumption since the individual isolate parameter estimates displayed positively skewed distributions and these parameters should always have positive values. Between isolate variability in *E*_max_ was assumed to be normally distributed since the individual parameter estimates displayed a symmetric distribution. Between isolate variability for *E*_0_ was not incorporated into the model.

Residual variability was modelled with additive and proportional components:
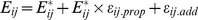
(3)
*E_ij_* and E^*^_ij_ are the observed and predicted normalised percentage uptake of hypoxanthine, respectively, and the residual variability components ε_i,j, prop_ and ε_i,j,add_ were assumed to be normally distributed with mean zero and the respective variances σ^2^_prop_ and σ^2^_add_. The nonlinear mixed-effects analysis was performed in NONMEM Version 7.1 (Icon Development Solutions, Ellicott City, MD, USA) in conjunction with the pearl-speaks-NONMEM program (PsN Version 3.5.3; [16], [17]), and the corresponding results were compared with the results obtained from the STS approach.

Lastly, a second nonlinear mixed-effects analysis was performed to estimate the effect of genotype grouping on the parameters, *EC*_50_ and γ. Equations 2 and 3 were used again to analyse data from all isolates simultaneously, but with the following modifications to equation 2:
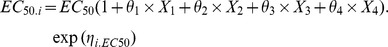


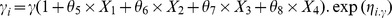

*X_1_–X_4_* are binary indicators (0 = no, 1 = yes) for an isolate with a single copy with mutation at position 86Y (*X_1_*), single copy with mutation at position 1042D (*X_2_*), two copies (all wild types; *X_3_*) or three or more copies (all wild types; *X_4_*), respectively, and *θ_1_–θ_4_* represent the difference in *EC*_50_ between the groups indicated by *X_1_–X_4_* (respectively) and the group with a single copy with wild type alleles at positions 86N and 1042N. A similar description can be made for *θ_5_–θ_8_* for *γ*.

### Simulation Study Comparing Methods for Detecting in vitro Correlates

To compare the STS and nonlinear mixed-effects modelling approaches for detecting genotype grouping effects on *EC_50_* in studies with smaller sample size, a simulation study was performed for mefloquine and lumefantrine (these two anti-malarials were selected because they vary in terms of the magnitude of the effect of *pfmdr1* copy number on *EC_50_*). To do this a random sample of 25, 50 or 100 isolates was drawn from the entire data set available for each of these drugs. For these isolates, the mean *EC_50_* (derived from either STS or nonlinear mixed-effects modelling) was compared between isolates that displayed a single copy (genotype1, above) versus two or more copies (genotypes 4 and 5 above). The prevalence of two or more copies was 39% for the population of isolates for mefloquine and 33% for the lumefantrine isolates.

This process was repeated 100 times, and was considered a “pseudo-simulation” study since step 1 involved drawing random samples from the observed data rather than simulating the data from a statistical model. The proportion of runs where the genotype grouping effect on *EC_50_* was detected (via the likelihood ratio test and defined as a p-value <0.05) was compared between the nonlinear mixed-effects modelling and STS approaches for the specified number of isolates (i.e. 25, 50 or 100).

## Results

In total 490 isolates were available for statistical analysis of at least one of the anti-malarial drugs tested. Concentration-effect data could be derived from a total of 421 (86%) isolates for chloroquine, 460 (94%) isolates for mefloquine, 324 (66%) isolates for lumefantrine, and 474 (97%) isolates for artesunate. The median number of observations per isolate across all drugs was 22 [range 7 to 44].

### Comparison of Standard Two-stage Method and Nonlinear Mixed-effects Modelling

Comparison of the two analytical approaches (STS method and nonlinear mixed-effects modelling) demonstrated that overall the population derived estimates of *EC_50_* and slope were similar (see Table 1). Nonlinear mixed-effects modelling yielded slightly lower between-isolate standard deviations (SDs) of the estimates of *EC_50_* and slope, with the exception of artesunate, where the SDs were marginally higher.

**Table 1:** Distribution of *EC_50_* and slope (γ) values for concentration-effect curves of chloroquine, mefloquine, lumefantrine and artesunate, derived from the two statistical modelling approaches: standard two-stage method and nonlinear mixed-effects modelling.

		*EC_50_*				Slope			
Anti-malarialdrug	Method	Estimate(nM)	95% referencerange	Estimate(log_e_ nM)	SD(log_e_ nM)	Estimate	95% referencerange	Estimate(log_e_ units)	SD(log_e_ units)
Chloroquine (n = 421)	STS	230.3†	63.8, 831.3‡	5.44	0.66	3.75†	1.47, 9.53‡	1.32	0.48
	NLME	240.7	68.6, 844.0*	5.48	0.64	4.14	1.85, 9.25*	1.42	0.41
Mefloquine (n = 460)	STS	67.2†	10.0, 450.5‡	4.21	0.97	2.82†	1.07, 7.45‡	1.04	0.50
	NLME	70.4	11.3, 435.6*	4.25	0.93	3.10	1.39, 6.92*	1.13	0.41
Lumefantrine (n = 324)	STS	38.6†	6.4, 234.6‡	3.65	0.92	2.47†	0.86, 7.09‡	0.90	0.54
	NLME	40.7	7.2, 228.0*	3.71	0.88	2.73	1.22, 6.10*	1.00	0.41
Artesunate (n = 474)	STS	2.75†	0.48, 15.7‡	1.01	0.89	5.85†	1.76, 19.46‡	1.77	0.61
	NLME	2.58	0.44, 15.32*	0.95	0.91	5.86	1.70, 20.10*	1.77	0.63

STS – standard two-stage method; NLME – nonlinear mixed-effects modelling; SD – standard deviation for between-isolate variability.

† Geometric mean.

‡ Calculated from estimated mean & SD (log_e_ scale) and converted to original scale.

* 95% prediction interval.

The proportion of *EC_50_* estimates from the STS approach with a coefficient of variation (CV) >15% was 15.4% (65/421) for chloroquine, 16.1% (74/460) for mefloquine, 24.4% (79/324) for lumefantrine and 27.9% (132/474) for artesunate. Isolates with a higher CV had significantly higher *EC_50_* values compared to those with CVs below or equal to the 15% threshold (see Table 2).

**Table 2:** Distribution of *EC_50_* values derived from the standard two-stage method for concentration-effect curves of chloroquine, mefloquine, lumefantrine and artesunate.

Anti-malarialdrug	Coefficient of variation ofisolate-specific estimates of *EC_50_*	Number ofisolates (%)			*EC_50_* estimate(nM)†		95% referencerange‡		p-value		
Chloroquine (n = 421)	CV <15%	356 (84.6)		220.0			67.7, 715.1		0.001		
	CV ≥15%	65 (15.4)		295.4			55.1, 1585.0				
Mefloquine (n = 460)	CV <15%	386 (83.9)		63.7			10.8, 374.9		0.007		
	CV ≥15%	74 (16.1)		88.8			7.9, 994.5				
Lumefantrine (n = 324)	CV <15%	245 (75.6)		34.9			6.9, 176.8		<0.001		
	CV ≥15%	79 (24.4)		52.7			6.0, 466.7				
Artesunate (n = 474)	CV <15%	342 (72.1)		2.42			0.43, 13.45		<0.001		
	CV ≥15%		132 (27.9)			3.87		0.76, 19.68			

CV – coefficient of variation;

† Geometric mean.

‡ Calculated from estimated mean & SD (log_e_ scale) and converted to original scale.

### Estimation of In Vitro Molecular Correlates Using Nonlinear Mixed-effects Modelling

Of the 490 isolates available for statistical analysis, the prevalence of the *pfmdr1* genotype groupings was 49% for single copy wild type (Genotype 1), 5% for single copy number with mutation at position 86Y (Genotype 2), 5% for single copy number with mutation at either positions 1042D (Genotype 3), and 26% for amplification with two copies (Genotype 4) and 15% for three or more copies (Genotype 5). The prevalence for mutation at position 1034C was 3% (5 isolates out of 187).

#### Single copy number with mutation at position 86Y

**Figure 1:**
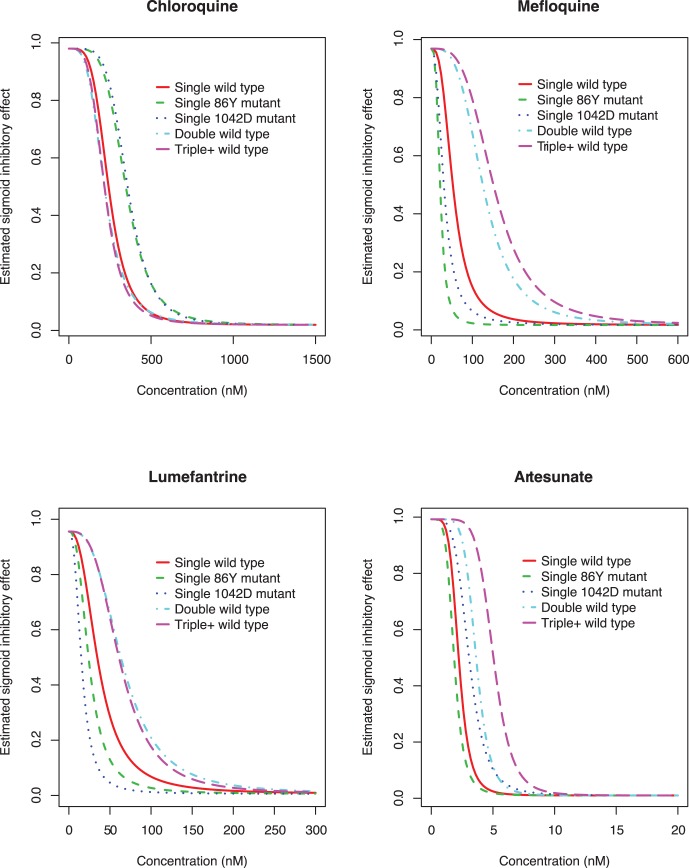
Estimated concentration-effect relationship (via nonlinear mixed-effects modelling) for chloroquine, mefloquine, lumefantrine and artesunate, by genotype grouping.

Isolates with a single copy number with mutation at position 86Y were associated with a decrease in the *EC_50_* value for mefloquine, lumefantrine and artesunate, compared to those with single copy wild type alleles 86N and 1042N. The magnitude of this decrease ranged from 59% for mefloquine, a 31% reduction for lumefantrine and a 17% reduction for artesunate (see Table 3, Figure 1). Conversely, a single copy number with mutation at position 86Y was associated with a relative increase of 44% for the *EC_50_* value of chloroquine. In contrast the slope of the effect-concentration curve did not differ significantly for these molecular comparisons (File S2).

**Table 3:** Effect of genotype grouping of *Pfmdr1* on *EC_50_* for chloroquine, mefloquine, lumefantrine and artesunate using nonlinear mixed-effects modelling.

	Genotype 1	Genotype 2	Genotype 3	Genotype 4	Genotype 5
	Single Copy	Single Copy	Single Copy	Double Copy	Triple+ Copy
*EC_50_*	WT^†^	86Y^‡^	1042D^‡^	86N/1042N^‡^	86N/1042N^‡^
Chloroquine ^#^					
Percent change	Reference category	44 (14,73)	48 (−4,100)	−10 (−23, 3)	−10 (−28, 7)
Estimated value (nM)	242 (223, 260)	347 (275,419)	359 (233, 483)	217 (184, 248)	217 (174, 260)
No. of isolates	212	20	19	113	57
Mefloquine					
Percent change	Reference category	−59 (−72, −46)	−42 (−67, −17)	139 (102,175)	188 (126,250)
Estimated value (nM)	53.0 (48.0, 58.1)	21.7 (14.7,28.7)	30.9 (17.6,44.1)	126.3 (107.0,145.9)	152.6 (119.8,185.4)
No. of isolates	230	25	24	118	63
Lumefantrine					
Percent change	Reference category	−31 (−62,0)	−57 (−76, −37)	82 (46,119)	75 (28,122)
Estimated value (nM)	35.7 (31.4,39.9)	24.6 (13.6,35.7)	15.5 (8.5,22.5)	65.0 (51.8,78.1)	62.4 (45.6,79.2)
No. of isolates	183	16	17	83	25
Artesunate^?^					
Percent change	Reference category	−17 (−39,6)	38 (−27,102)	63 (35,92)	127 (74,169)
Estimated value (nM)	2.3 (2.1,2.6)	1.8 (1.3,2.3)	3.1 (1.6,4.4)	3.6 (3.1,4.2)	4.9 (3.9,6.2)
No. of isolates	234	24	24	123	69

95% confidence intervals in brackets; ^†^Reference group; ^#^ *E_max_* fixed to 0.98; ^?^ *E_0_* fixed to 0.01.

Between−isolate variance estimate (standard errors) for *EC_50_*: − 0.39(0.026) chloroquine, 0.56(0.043) mefloquine, 0.63(0.050) lumefantrine, 0.67(0.048) artesunate.

Within-isolate variance estimates (standard errors) are:-.

proportional – 0.013(0.0018) chloroquine, 0.010(0.0011) mefloquine, 0.019(0.0020) lumefantrine, 0.025(0.0037) artesunate;

additive – 0.001(0.0002) chloroquine, 0.001(0.0001) mefloquine, 0.0009(0.0002) lumefantrine, 0.0007(0.0002) artesunate.

#### Single copy number with mutation at position 1042D

For mefloquine and lumefantrine, isolates with a single copy number with mutations at positions 1042D (Genotype 3) were associated with a decrease in the *EC_50_* value compared with single copies of wild type (Genotype 1). The magnitude of this decrease was 42% for mefloquine (population mean estimate of 30.9 versus 53.0 nM) and 57% for lumefantrine (population mean estimate of 15.5 versus 35.7 nM) (see Table 3, Figure 1). Conversely, Genotype 3 variants were associated with a relative increase of 48% for the *EC_50_* value of chloroquine (population mean estimate of 359 versus 242 nM). For artesunate a relative increase in the *EC_50_* estimate was also observed, however, the 95% confidence interval ranged from a relative decrease of 27% to a relative increase of 102%. As with the variants at codon 86Y, minimal changes in the slope of the concentration-effect profile with the 1042D variant were observed (File S2).

#### Pfmdr1 Amplification

Isolates with two or more copies of *pfmdr1* were all “wild type” (86N and 1042N). Those with two or three-or-more copies had significantly higher *EC_50_* values for mefloquine (139% and 188%, respectively), lumefantrine (82% and 75%, respectively) and artesunate (63% and 127%, respectively) compared with isolates with a single copy wild type (see Table 3, Figure 1). In contrast the mean *EC_50_* for chloroquine was similar for isolates irrespective of their copy numbers (Table 3). Compared to single copy isolates, those with two or more copies had steeper slopes of the concentration-effect curves for mefloquine, lumefantrine and artesunate, whereas they were marginally shallower in the chloroquine assay (File S2).

### Simulation Study Comparing Standard Two-stage and Nonlinear Mixed-effects Methods for Detecting in vitro Correlates

To assess the power of detecting significantly important shifts in the dose response curve associated with specific parasite genotypes a pseudo-simulation study was conducted using hypothetical study samples of 25, 50 or 100 isolates from a population of wild type isolates where the prevalence of two or more copies was 39% for mefloquine datasets and 33% for lumefantrine datasets.

For both mefloquine and lumefantrine the proportions of studies where the shift associated with genotype was significant was similar for both statistical approaches (see Table 4). As expected the statistical power increased with study size (number of isolates). The effect of *pfmdr1* amplification was more readily apparent for mefloquine, where almost studies of all sample sizes as low as 25 were able to detect a statistically significant relationship. The magnitude of the shift associated with *pfmdr1* amplification was lower with lumefantrine (1.8 fold vs 2.6 for mefloquine) in the population of isolates, and a corresponding higher sample size (minimum 100 isolates) was required to detect a genotype effect.

**Table 4:** Results from the pseudo-simulation study comparing the standard two-stage and nonlinear mixed-effects modelling approaches for detecting a binary genotype grouping effect on *EC_50_* in studies with 25, 50 or 100 isolates.

Anti-malarial	Analyticalapproach	Number of IsolatesAssessed		
		25	50	100
**Mefloquine** †,‡	**STS**	85%?	100%	100%
	**STS (CV** **≤15%)◊**	85%	98%	100%
	**NLME**	87%	100%	100%
**Lumefantrine** † *	**STS**	49%	77%	98%
	**STS (CV** **≤15%)◊**	42%	64%	93%
	**NLME**	46%	74%	97%

STS - standard two-stage; NLME - nonlinear mixed-effects modelling; CV – coefficient of variation.

? Percentage of runs where shift in *EC_50_* was detected according to parasite genotype via the likelihood ratio test.

† Genotype was grouped as a single wild type (reference category) *versus* wild type with two or more copies.

‡ For the entire dataset (the assumed population) of isolates with wild type alleles exposed to mefloquine (230 (50%) isolates with a single copy & 181 (39%) with two or more copies), the estimated genotype effect on *EC_50_* was 2.60 (ratio of geometric means; 95% CI 2.24 to 3.02) from the standard two-stage approach and 2.55 fold (95% CI 2.32 to 2.78) from the nonlinear mixed-effects modelling approach.

* For the entire dataset (the assumed population) of isolates with wild type alleles exposed to lumefantrine (183 (56%) isolates with a single copy & 108 (33%) with two or more copies), the estimated genotype effect on *EC_50_* was 1.91 (ratio of geometric means; 95% CI 1.57 to 2.32) from the standard two-stage approach and 1.82 fold (95% CI 1.43 to 2.22) from the nonlinear mixed-effects modelling approach.

◊ Only includes those isolates where the coefficient of variation (CV) of the *EC_50_* estimate in the first stage of the STS analysis was ≤15% (∼84% of total sample for mefloquine & ∼76% for lumefantrine).

When an exclusion criterion based on the precision of the *EC_50_* was applied (CV>15%), the statistical power of the STS fell below that of the nonlinear mixed-effects modelling in which all isolates were analysed in the model simultaneously. This loss in statistical power was not observed for mefloquine where the relative effect size of genotype group on *EC_50_* was 2.6-fold but was observed for lumefantrine which had an effect size of 1.8 fold; the absolute loss in statistical power ranged from 4 to 10% for sample sizes of 25, 50 and 100.

### Statistical Power for Future in vitro Studies

**Figure 2:**
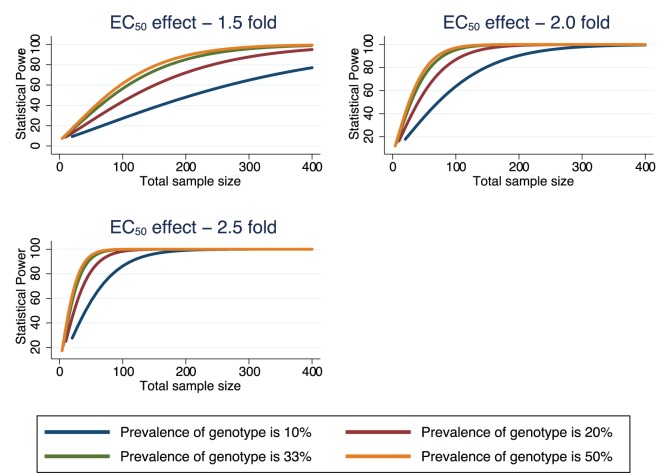
Statistical power by sample size for a comparison of *EC_50_* (geometric mean) between two genotypes.

Figure 2 presents the statistical power calculated using formulae for a two-sample comparison of the geometric mean of *EC_50_* between two genotype groups. The figure highlights that for genotype groups with a low prevalence (e.g. 10%, similar to what was observed in this study population for single copy with mutation at positions 86Y or 1042D) a larger number of isolates would be required to achieve 80% statistical power for detecting a genotype effect of 1.5 fold (430 total isolates required of which 43 will have genotype group1 and 387 genotype group2), 2 fold (150 total isolates) and 2.5 fold (80 total isolates). As the prevalence of genotype group increases the total sample size required to achieve 80% statistical power decreases, however, to detect a genotype effect of 1.5 fold (which may be of clinical importance) 245 isolates are required if the prevalence of genotype is 20%, 174 isolates if prevalence is 33% and 156 isolates if the prevalence is 50%.

## Discussion

The primary aim of *in vitro* drug susceptibility testing of Plasmodia is to derive estimates of drug activity independent from the host and pharmaceutical factors known to confound *in vivo* efficacy studies. Several *in vitro* approaches have been established and validated although all remain vulnerable to a variety of methodological factors that are manifest in inherent within- and between- assay variability. In the current study data from a large number of clinical isolates of *P. falciparum* from the western border of Thailand were used to define the correlation of *in vitro* drug susceptibility of four antimalarial drugs with polymorphisms of the *pfmdr1* gene, known to be a key determinant of multidrug resistance [11], [12]. Our results provide precise estimates of the shifts in drug susceptibility confirming previous observations that single nucleotide polymorphisms of *pfmdr1* are associated with decreased susceptibility to chloroquine but increased susceptibility to artesunate, mefloquine and lumefantrine. Whereas increased *pfmdr1* copy number was associated with reduced susceptibility to artesunate, mefloquine and lumefantrine.

The main focus of the study was to compare two statistical approaches for estimating drug susceptibility parameters and assess their relative merits for defining between-isolate variability and validating putative molecular markers of antimalarial drug resistance. Our results demonstrate that the STS approach and nonlinear mixed-effects modelling provided very similar population estimates of *EC_50_*s, however the STS was particularly vulnerable to exclusion of isolates with poor precision. Between 15–28% of isolates were excluded from the STS analysis and since these isolates were more likely to be highly resistant isolates, the inevitable consequence was an under-estimation of the population mean *EC_50_*.

The *in vitro* data used in this study were derived from rich balanced designs compared to the sparse and unbalanced designs commonly observed for anti-malarial pharmacokinetic studies [18]–[20]. Separation of the within and between-isolate variance components in the nonlinear mixed-effects model provided minimal reductions in the between-isolate variability for the model parameters of three of the four drugs assessed, therefore the method did not result in greater statistical power for detecting the genotype effect on *EC_50_*.

In the nonlinear regression analyses using the STS approach the lower precision of the *EC_50_* estimates was likely an artefact of the design of the *in vitro* experiment in which doubling dilutions of drug concentrations is applied, resulting in fewer measurements and larger intervals at higher drug concentrations [5]. Ideally such isolates should be retested with a higher series of drug dilutions. However, without culture adaptation of the parasite this is usually not feasible. Instead such isolates are often simply excluded from the STS approach, with inevitable reduction in the estimate of the overall population *EC_50_* and the possibility of missing the early detection of resistance emergence. Although nonlinear mixed-effects modelling cannot overcome inclusion criteria for the assay itself, it does offer advantages over the STS approach by including all isolates in the analysis simultaneously to ensure each isolate contributes some information towards estimation of the parameters. In this approach the within-isolate variability is determined, thus, drug susceptibility estimates are derived after controlling for lab covariates such a parasite staging, assay duration and different batches of drug plates [21]. Further, those isolates with less reliable individual data (e.g. resistant isolates with no measurements between the concentrations associated with maximum and minimum effect) do not contribute the same amount of information as other isolates (i.e. not all isolates are weighted equally in the model). Conversely the STS approach weights the individual isolate-specific estimates equally. This can be problematic since it has been shown in the separate isolate-specific nonlinear regressions (i.e. stage 1 of the analysis which takes no account of the other isolates in the population) that the most resistant isolates tend to have *EC_50_*s overestimated as curve fitting estimates the *EC_50_* to be approximately the mid-point between two concentrations with very different effect measurements [5].

The nonlinear mixed-effects modelling approach allowed a comprehensive analysis of the *in vitro* – molecular correlation for four major antimalarial drugs. Our findings confirm the role of *pfmdr1* copy number and reduced drug susceptibility to mefloquine, lumefantrine and artesunate [10]–[12], [22], the effect ranging from 63 to 188%. Whereas an increase from 2 to 3 or more copies resulted in further increments of mefloquine and artesunate this was not apparent for lumefantrine. We also estimated the change in both the *EC_50_* and slope of the effect-concentration curve due to single nucleotide polymorphisms at 86Y and 1042D. These mutations occurred almost entirely in isolates with a single copy of *pfmdr1*, and were associated with a modest increase in the *EC_50_* of chloroquine (44 to 48%). Such estimates can be used for predictions of isolate specific effect-concentration profiles for different genotype groups, and included in within-host pharmacokinetic-pharmacodynamic models [23] and population level anti-malarial resistance models [24].

Our results highlight the challenges of validating candidate markers of drug resistance. The power to detect statistical significance depends upon the shift in the dose response curve, and the prevalence of the genotype in the population. There may be a tendency to dismiss important correlates that are confounded by inadequate sample size or sample bias. Our sample size calculations demonstrate that in order to achieve 80% power to detect an *in vitro* – molecular effect of a genetic mutation with a prevalence of 10% and a 1.5 fold shift in the *EC_50,_* a sample size of 430 isolates would be required. Whereas a mutation with a greater effect such as *pfmdr1* copy number on mefloquine sensitivity would be detected with a much smaller sample size (eg only 25 isolates required for an effect size of 2.5 fold and a prevalence of genotype in the population of 50%).

In conclusion, our comparison of the conventional STS approach versus the more sophisticated nonlinear mixed-effects modelling, for the analysis of *in vitro* drug susceptibility data, did not demonstrate any major advantage of applying nonlinear mixed-effects modelling providing data were generated from multiple drug concentrations around the *EC_50_*, over the most dynamic part of the dose response curve. However, when sporadic isolates with high resistance and low precision were present within a parasite population the nonlinear mixed-effects model estimates were likely to be less biased, and provide greater power at detecting *in vitro* - molecular correlates of candidate molecular markers of drug resistance.

## Supporting Information

File S1 ***In vitro*** **drug susceptibility data and** ***pfmdr1*** **polymorphisms of all isolates included in the analysis.** Drug susceptibility data presented were generated from the standard two-stage regression analysis.
(XLSX)

File S2 **Effect of genotype grouping of** ***Pfmdr1*** **on slope values for chloroquine, mefloquine, lumefantrine and artesunate using nonlinear mixed-effects modeling.**
(PDF)
