# Supplementary material for: Nonlinear Mixed-Effects Modelling of In Vitro Drug Susceptibility and Molecular Correlates of Multidrug Resistant Plasmodium falciparum
Source: PLoS One. 2013 Jul 24;8(7):e69505. doi: 10.1371/journal.pone.0069505 (PMC3722116; doi:10.1371/journal.pone.0069505)
Supplement: File S2 — Effect of genotype grouping of Pfmdr1 on slope values for chloroquine, mefloquine, lumefantrine and artesunate using nonlinear mixed-effects modeling. (PDF) [file pone.0069505.s002.pdf]

Supplementary file S2: Effect of genotype grouping of *Pfmdr1* on slope values* for chloroquine, mefloquine, lumefantrine and artesunate using nonlinear mixed-effects modelling

**Slope**	Genotype 1 Single Copy WT [†]	Genotype 2 Single Copy 86Y [‡]	Genotype 3 Single Copy 1042D [‡]	Genotype 4 Double Copy 86N/1042N[‡]	Genotype 5 Triple+ Copy 86N/1042N[‡]
Chloroquine [#]					
Percent change	Reference category	12 (-10, 33)	22 (-2, 46)	-14 (-23, -6)	-5 (-17, 6)
Estimated value	4.2 (4.0, 4.5)	4.7 (3.8, 5.7)	5.2 (4.2, 6.2)	3.6 (3.3, 4)	4.0 (3.5, 4.5)
No. of isolates	212	20	19	113	57
Mefloquine					
Percent change	Reference category	14 (-5, 32)	-14 (-31, 4)	21 (10, 32)	23 (6, 39)
Estimated value	2.9 (2.7, 3.0)	3.3 (2.7, 3.8)	2.5 (2.0, 3.0)	3.5 (3.2, 3.8)	3.5 (3.1, 4.0)
No. of isolates	230	25	24	118	63
Lumefantrine					
Percent change	Reference category	4 (-8, 17)	3 (-13, 19)	17 (2, 31)	25 (6, 44)
Estimated value	2.6 (2.4, 2.8)	2.7 (2.4, 3.0)	2.7 (2.3, 3.1)	3.0 (2.6, 3.4)	3.2 (2.7, 3.7)
No. of isolates	183	16	17	83	25
Artesunate [^]					
Percent change	Reference category	-7 (-27, 12)	-11 (-38, 16)	29 (11, 47)	33 (12, 53)
Estimated value	5.2 (4.8, 5.6)	4.8 (3.8, 5.8)	4.6 (3.2, 6.0)	6.7 (5.8, 7.6)	6.9 (5.8, 7.9)
No. of isolates	234	24	24	123	69

95% confidence intervals in brackets; [†]Reference group; #E_{max} fixed to 0.98; ^E_0 fixed to 0.01

Between-isolate variance estimate (standard errors) for slope:- 0.15(0.019) chloroquine, 0.15(0.015) mefloquine, 0.16(0.022) lumefantrine, 0.33(0.042) artesunate

Within-isolate variance estimates (standard errors) are:-

proportional – 0.013(0.0018) chloroquine, 0.010(0.0011) mefloquine, 0.019(0.0020) lumefantrine, 0.025(0.0037) artesunate;

additive – 0.001(0.0002) chloroquine, 0.001(0.0001) mefloquine, 0.0009(0.0002) lumefantrine, 0.0007(0.0002) artesunate.
